# CircRNA-encoded protein fine-tunes ROS homeostasis and engages conserved JAK-STAT antiviral defenses in *Drosophila*

**DOI:** 10.1128/jvi.01708-25

**Published:** 2025-12-22

**Authors:** Dongyang Guo, Wen Xu, Liqin Zhang, Ting Cui, Liqin Tang, Qingfa Wu

**Affiliations:** 1 Department of Pharmacy, The First Affiliated Hospital of USTC, Division of Life Sciences and Medicine, University of Science and Technology of China 12652, Hefei, Anhui, China; 2 Key Laboratory of Anhui Province for Emerging and Reemerging Infectious Diseases, University of Science and Technology of China 12652, Hefei, China; 3 Key Laboratory of Aging and Cancer Biology of Zhejiang Province, School of Basic Medical Sciences, Hangzhou Normal University 26494, Hangzhou, China; Wageningen University & Research, Wageningen, Netherlands

**Keywords:** antiviral immunity, CircRNA-encoded protein, ROS, MAPK cascade, JAK-STAT pathway, *Drosophila *C virus, *Drosophila melanogaster*

## Abstract

Multicellular organisms rely on multilayered immune systems to defend against pathogen invasion. In *Drosophila melanogaster*, the primary antiviral barrier is RNA interference (RNAi). However, many viruses encode suppressors that disable RNAi, forcing hosts to activate complementary defense strategies. One such strategy involves the circular RNA circZfh1, which encodes the protein CRAV. CRAV is essential for activating the JAK-STAT pathway and providing antiviral protection when RNAi is neutralized. However, the molecular mechanism linking CRAV expression to *upd3* induction and JAK-STAT activation remains unclear. Here, we show that CRAV directly interacts with the Ca²^+^-binding domain of the NADPH oxidase Nox, enhancing its enzymatic activity. This interaction promotes the generation of moderate reactive oxygen species (ROS) that act as signaling intermediates rather than stress inducers. CRAV-induced ROS selectively activate the ASK1-p38 mitogen-activated protein kinase cascade, which in turn triggers JAK-STAT signaling and the expressions of antiviral cytokines and effectors. Loss of Nox or inhibition of ASK1-p38 abolishes CRAV-mediated protection, underscoring the necessity of finely tuned redox signaling. These findings reveal a direct mechanistic link between a circRNA-derived protein and conserved innate immune pathways, highlighting the pivotal role of controlled ROS signaling in antiviral defense.

**IMPORTANCE:**

Antiviral immunity depends on the balance between host defenses and viral countermeasures. In fruit flies, RNA interference (RNAi) represents the primary barrier to viral infection, but viruses often disable this pathway. We show that the circRNA-encoded protein CRAV provides a backup defense by directly binding the NADPH oxidase Nox to generate moderate reactive oxygen species (ROS). Unlike damaging oxidative stress, these ROS serve as signaling cues that activate p38 and JAK-STAT pathways, which in turn drive antiviral cytokine production. This study uncovers how a circRNA-derived protein engages conserved redox-sensitive immune signaling, illustrating an adaptive strategy that ensures protection when RNAi is compromised. The results provide fundamental insights into the evolutionary diversification of circRNA-encoded proteins and broaden our understanding of how finely tuned ROS signaling contributes to innate antiviral immunity.

## INTRODUCTION

Viruses and their hosts are engaged in a constant evolutionary arms race, with hosts developing diverse antiviral defenses and viruses evolving countermeasures to overcome them (1, 2). In *Drosophila melanogaster*, the primary innate antiviral defense relies on RNA interference (RNAi), where viral double-stranded RNAs are processed into small interfering RNAs (siRNAs) that guide Argonaute-2 to degrade viral transcripts (3, 4). However, many RNA viruses encode viral suppressors of RNAi (VSRs) that effectively disable this frontline response, forcing hosts to evolve complementary antiviral strategies (5–7). Beyond RNAi, *Drosophila* shares several conserved innate immune signaling pathways with mammals—including JAK-STAT, Toll, Imd, and cGAS-STING—that orchestrate cytokine production, stress responses, and apoptosis to restrict viral replication (8–16). Recent studies have demonstrated that when RNAi is compromised, *Drosophila* can activate secondary immune programs, such as noncanonical immune pathway, stress-induced transcriptional responses, and cytokine-mediated signaling, to counteract viral infection (6, 17, 18).

Reactive oxygen species (ROS) were historically considered toxic metabolic byproducts that damage macromolecules and promote cell death. However, accumulating evidence demonstrates that ROS also function as versatile signaling intermediates that fine-tune immune responses to infection (19, 20). Depending on their intensity, duration, and subcellular origin, ROS can either enhance antiviral defenses or be co-opted to promote viral persistence (19, 21–28). In mammals, moderate ROS levels often support innate immunity. Dengue virus infection requires balanced ROS levels since depletion weakens antiviral responses while controlled ROS promote immune signaling (23). In contrast, viruses such as SARS-CoV-2 or Zika virus suppress antioxidant pathways, leading to oxidative stress that dampens interferon signaling and enhances replication (27–31). Hepatitis viruses also manipulate ROS to support replication or block cGAS-STING activation (32–36).

In *Drosophila,* ROS act in a context-dependent way. At epithelial surfaces such as the gut, dual oxidase (Duox)-derived ROS form a rapid antimicrobial barrier that complements antimicrobial peptides (37). At the systemic level, ROS activate extracellular signal-regulated kinase (ERK) and p38 mitogen-activated protein kinase (MAPK) pathways that drive cytokine secretion and JAK-STAT signaling (38–40). The gut-enriched circATP8B2 regulates Duox-dependent ROS to enhance antiviral protection (41). Loss of carbonyl reductase Sniffer (Sni) elevates ROS, which disrupts blood-brain barrier integrity and permits Sindbis virus invasion of neural tissue (42). These studies highlight a general principle in which basal or moderate ROS strengthen antiviral signaling, whereas excessive ROS damage host tissue and favor viral pathogenesis. MAPK cascades represent key redox-sensitive modules. Among the three MAPKs in *Drosophila*—ERK, c-Jun N-terminal kinase (JNK), and p38—the p38 pathway is most sensitive to ROS (43). In mammals, p38 regulates cytokine production, apoptosis, and interferon responses (44). In flies, p38 also mediates defense against bacteria, fungi, and viruses (45). Infection with the DNA virus IIV-6 induces ROS that activate p38b, which triggers Upd secretion and JAK-STAT signaling (40). These findings establish ROS-MAPK signaling as a conserved antiviral mechanism across kingdoms.

A striking example of host counter-defense in *Drosophila* was recently uncovered by Guo et al. (46). Infection with *Drosophila C virus* (DCV), a natural *Drosophila* pathogen that encodes the potent VSR DCV-1A, induces the expression of a circular RNA, *circZfh1*. Remarkably, *circZfh1* is not merely a noncoding transcript but encodes a 274-amino acid protein, CRAV. Due to a frameshift at the 3′ end, CRAV contains a unique 69-amino acid C-terminal extension that has undergone rapid evolutionary diversification across *Drosophila* species (46). CRAV expression is both necessary and sufficient for the antiviral function of *circZfh1*. Importantly, its activity is evident only against wild-type DCV harboring an intact VSR, but not against VSR-deficient mutants, suggesting that CRAV functions as a backup defense mechanism specifically mobilized when RNAi is neutralized. Mechanistically, CRAV promotes secretion of the cytokine Unpaired 3 (Upd3), which activates the JAK-STAT pathway through the receptor Domeless. This signaling cascade upregulates effector genes such as *TotA* and *socs36E*, enhancing host survival (46). Loss-of-function studies using CRISPR/Cas9 knockout of *circZfh1* confirmed its essential role as flies lacking CRAV showed impaired JAK-STAT activation and heightened susceptibility to DCV infection (46). Despite these insights, the molecular mechanism linking CRAV expression to *upd3* induction and JAK-STAT activation has remained elusive.

In this study, we delineate the molecular pathway by which the circRNA-derived protein CRAV activates antiviral immunity in *Drosophila*. We demonstrate that the evolutionarily novel C-terminal domain of CRAV interacts with the Ca²^+^-binding regulatory region of the NADPH oxidase Nox, enhancing its enzymatic activity. This interaction promotes the generation of moderate levels of ROS that act as signaling intermediates rather than inducers of oxidative stress. These Nox-derived ROS specifically activate the ASK1-p38 MAPK signaling cascade, which in turn drives JAK-STAT pathway activation and antiviral cytokine production. Together, our findings establish a direct mechanistic link from a circRNA-encoded peptide to conserved innate immune signaling and highlight the critical role of finely tuned ROS signaling in coordinating host antiviral defenses.

## RESULTS

### CRAV induces ROS accumulation in *Drosophila* cells

**Fig 1:**
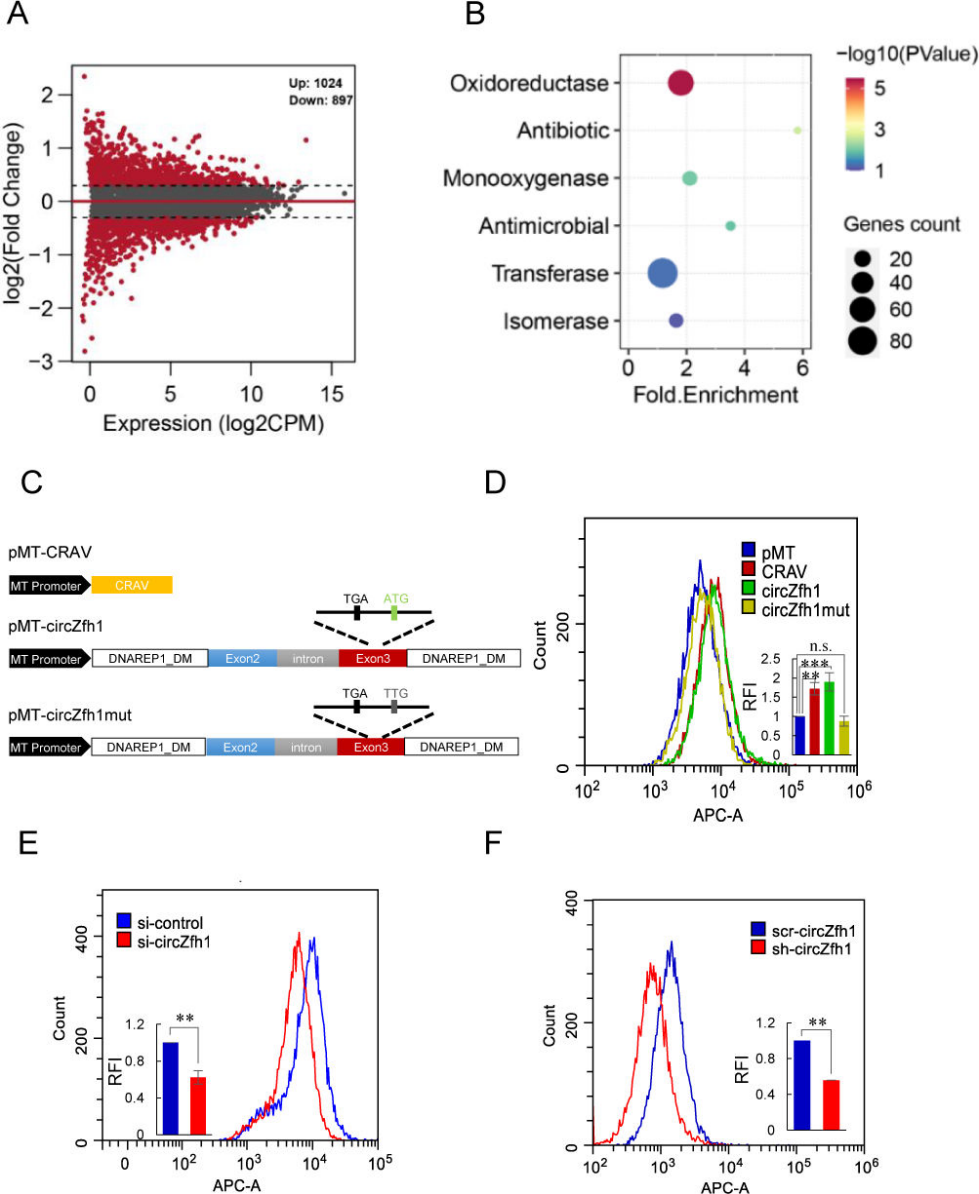
CRAV induces ROS accumulation in Drosophila cells. (**A**) S2 cells were transfected with the pMT-CRAV or the control empty pMT vector and induced with CuSO₄. After 24 h, cells were infected with DCV for 48 h and subjected to RNA-seq. Differentially expressed genes (DEGs) were defined using a log₂ fold-change threshold of 0.3, yielding 1,024 upregulated and 897 downregulated genes. (**B**) Functional enrichment analysis of upregulated genes in pMT-CRAV–transfected cells relative to pMT controls following 48 h of DCV infection. Dot color indicates the *P* value, dot size represents the number of genes per functional category, and the x-axis shows fold enrichment. (**C**) Schematic diagrams of exogenous expression constructs. pMT-CRAV: CRAV expression vector. pMT-circZfh1: circZfh1 expression vector, with the CRAV start (ATG) and stop (TGA) codons indicated. pMT-circZfh1mut: circZfh1 construct with the CRAV start codon mutated from ATG to TTG. (**D–F**) Flow cytometry analysis of intracellular ROS levels in S2 cells subjected to different treatments without DCV infection. (**D**) S2 cells transfected with pMT, pMT-CRAV, pMT-circZfh1, or pMT-circZfh1mut. (**E**) S2 cells transfected with si-circZfh1 or si-control. (**F**) Stable cell lines expressing scrambled shRNA (scr-circZfh1) or circZfh1-targeting shRNA (sh-circZfh1). Overlay histograms show fluorescence intensity, and bar graphs depict relative fold changes calculated from the mean fluorescence intensity (**D–F**). Data represent mean ± SD from three independent experiments (**D–F**). Statistical analysis was performed using one-way ANOVA (**D**), Student’s *t*-test (**E–F**): **P < 0.01; ***P < 0.001; n.s., not significant.

Having established that the circular RNA circZfh1 encodes the protein CRAV to activate the JAK-STAT pathway and confer antiviral immunity (46), we next sought to determine the upstream molecular mechanism by which CRAV initiates this signaling cascade. To investigate this, we ectopically expressed CRAV in *Drosophila* S2 cells and subsequently challenged them with DCV infection. Transcriptomic profiling revealed widespread transcriptional remodeling, with the expressions of 1,024 genes significantly upregulated and those of 897 downregulated compared to control cells (Fig. 1A). Gene ontology (GO) enrichment analysis of the upregulated genes highlighted strong enrichment for redox-related biological processes (Fig. 1B), implicating a role for CRAV in oxidative stress regulation.

We next examined whether CRAV alters intracellular ROS levels. S2 cells were transfected with expression constructs for pMT, pMT-CRAV, pMT-circZfh1, or circZfh1mut, which carries an ATG→TTG substitution that abolishes CRAV translation (Fig. 1C). Flow cytometry analysis showed that circZfh1 or CRAV expression significantly increased ROS levels, whereas circZfh1mut had no effect (Fig. 1D). To test whether endogenous circZfh1 contributes to ROS homeostasis, we used siRNAs targeting the back-splice junction of circZfh1, which specifically depletes circZfh1 without affecting Zfh1 mRNA or ZFH1 protein (46). Knockdown of circZfh1 markedly reduced ROS accumulation (Fig. 1E). Consistent results were obtained using stable S2 cell lines expressing shRNA against the same junction sequence (sh-circZfh1) compared with scrambled controls (scr-circZfh1) (Fig. 1F) (46). Taken together, these results demonstrate that CRAV is both necessary and sufficient for ROS in *Drosophila* cells.

### CRAV-induced ROS production is mediated by Nox

ROS can originate from multiple intracellular sources, most notably mitochondrial dysfunction or the activity of NADPH oxidases. Mitochondrial disruption can impair the electron transport chain, causing electron leakage and collapse of the mitochondrial membrane potential, which together contribute to increased ROS generation (47). To pinpoint the source of CRAV-induced ROS, we first tested whether CRAV expression perturbed mitochondrial integrity. Assessment of mitochondrial membrane potential revealed that neither CRAV expression nor circZfh1 knockdown altered mitochondrial function (Fig. S1A and B). This suggested that mitochondria are unlikely to be the primary source of CRAV-triggered ROS.

**Fig 2:**
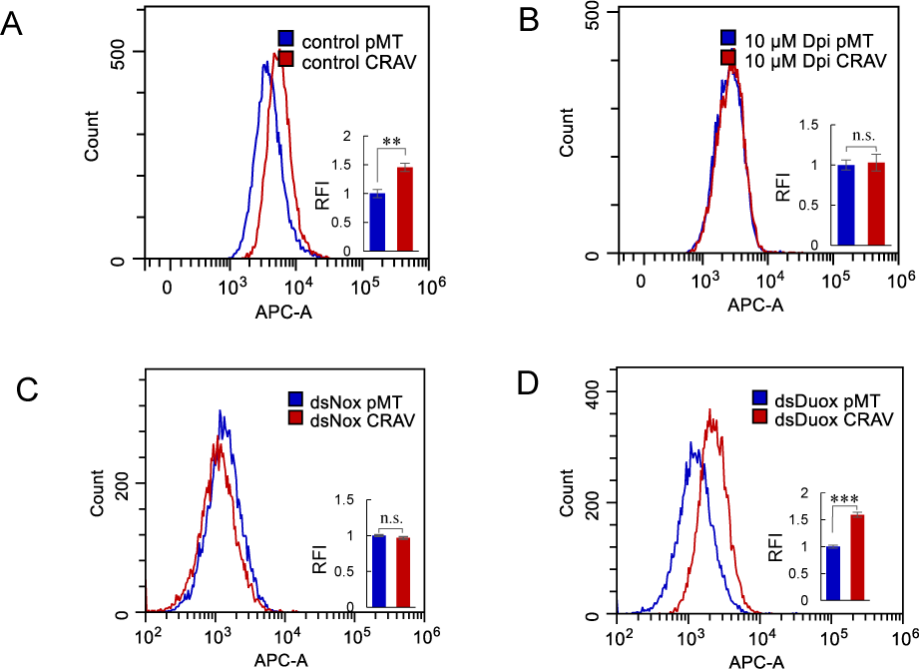
CRAV-induced ROS production is mediated by Nox. Flow cytometry analysis of intracellular ROS levels in S2 cells. (**A**) Cells pretreated with DMSO (control) or the NADPH oxidase inhibitor DPI. (**B**) Cells pretreated with 10 μM DPI for 1 h and then transfected with pMT or pMT-CRAV. (**C–D**) Cells pretreated with dsRNAs targeting Nox (**C**) or Duox (**D**) and transfected with pMT or pMT-CRAV. Overlay histograms show the fluorescence intensity, and bar graphs depict relative fold changes calculated from the mean fluorescence intensity (A–D). Data represent mean ± SD from three independent experiments (**A–D**). Statistical analysis was performed using Student’s *t*-test (**A–D**): **P < 0.01; ***P < 0.001; n.s., not significant.

We next examined whether NADPH oxidases contribute to this effect. S2 cells were treated with increasing concentrations of diphenyleneiodonium chloride (DPI), a widely used inhibitor of NADPH oxidase activity (48), and then transfected with either pMT or pMT-CRAV. Notably, the CRAV-mediated increase in ROS was completely abolished in the presence of DPI (Fig. 2A and B; Fig. S1A and B). In *Drosophila*, two NADPH oxidases, Duox and Nox, regulate distinct ROS-dependent physiological and immune processes (49, 50). To assess their involvement in CRAV-mediated ROS production, we performed targeted knockdown using dsRNA in S2 cells (Fig. S1E). Strikingly, silencing of Nox, but not Duox, significantly reduced ROS levels in CRAV-expressing cells (Fig. 2C and D; Fig. S1F). Together, these findings demonstrate that CRAV promotes ROS accumulation primarily through Nox-dependent NADPH oxidase activity, rather than via mitochondrial dysfunction.

### CRAV interacts with the Nox Ca²^+^-binding domain to potentiate ROS production

**Fig 3:**
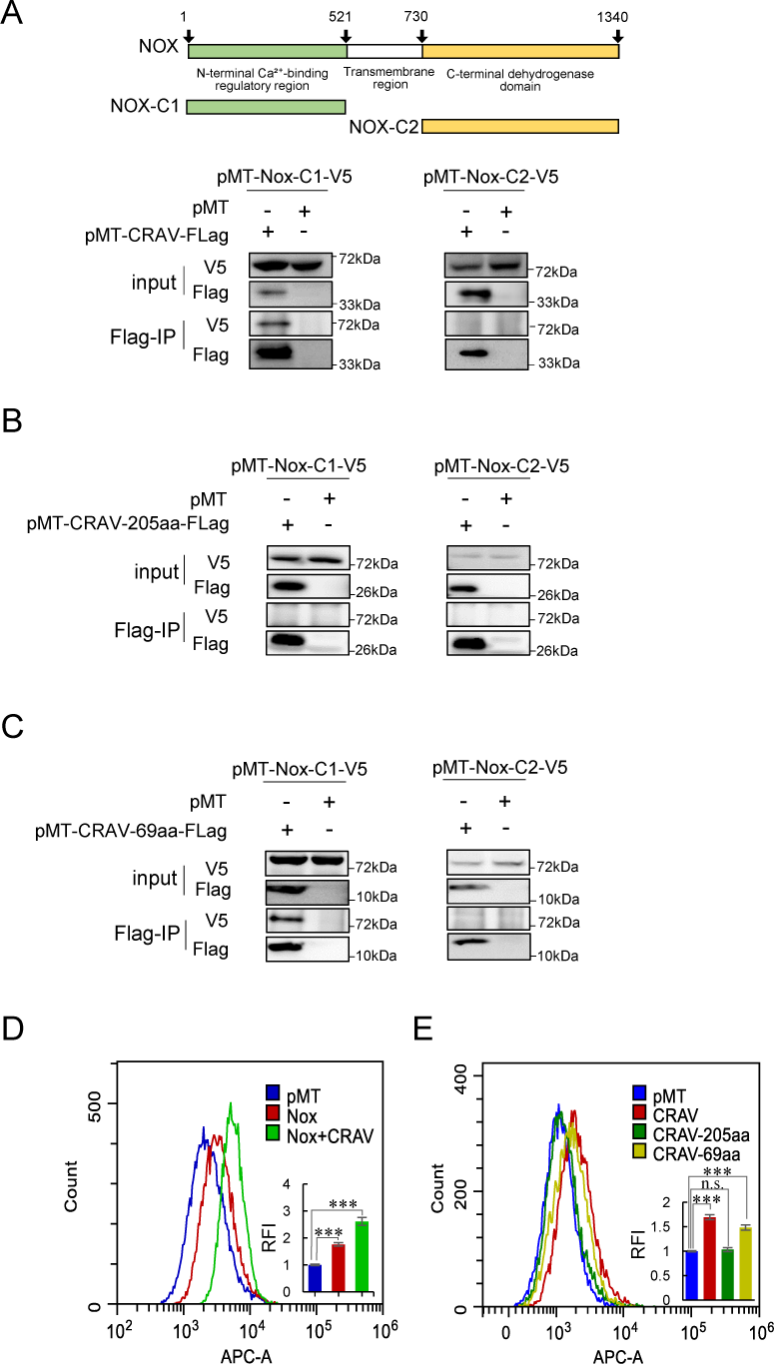
CRAV interacts with the Nox Ca²^+^-binding domain to potentiate ROS production. (**A**) Schematic representation of the two intracellular domains of Nox: the Nox-C1 and the Nox-C2 (upper panel). Co-IP analysis of S2 cells co-transfected with pMT-Nox-C1-V5 or pMT-Nox-C2-V5 and pMT-CRAV-Flag. Cell lysates were immunoprecipitated with anti-Flag antibody and analyzed by Western blot using anti-V5 and anti-Flag antibodies (lower panel). (**B**) Co-IP of S2 cells co-transfected with Nox-C1-V5 or Nox-C2-V5 and the N-terminal fragment of CRAV (pMT-CRAV-205aa-Flag). (**C**) Co-IP of S2 cells co-transfected with Nox-C1-V5 or Nox-C2-V5 and the C-terminal fragment of CRAV (pMT-CRAV-69aa-Flag). Immunoprecipitation and Western blot detection were performed as in (**A**). (**D, E**) Flow cytometry analysis of intracellular ROS levels. (**D**) S2 cells transfected with pMT or pMT-Nox-C1 and cells co-transfected with pMT-Nox and pMT-CRAV. (**E**) S2 cells transfected with pMT (empty vector), pMT-CRAV, pMT-CRAV-205aa, or pMT-CRAV-69aa. Overlay histograms show fluorescence intensity, and bar graphs depict relative fold changes calculated from the mean fluorescence intensity (**D, E**). Data represent mean ± SD from three independent experiments (**D, E**). Statistical analysis was performed using one-way ANOVA (**D, E**): ***P < 0.001; n.s., not significant. Representative results from three independent experiments are shown in (**A–C**).

*Drosophila* Nox comprises two key intracellular domains: an N-terminal Ca²^+^-binding regulatory region (Nox-C1) and a C-terminal dehydrogenase domain (Nox-C2), which contains FAD- and NADPH-binding sites (51–53). To determine whether CRAV directly interacts with Nox to regulate ROS levels, we cloned Nox-C1 and Nox-C2 separately into expression vectors and co-expressed them with Flag-tagged CRAV in S2 cells. Co-immunoprecipitation (Co-IP) analysis revealed a specific interaction between CRAV and the Nox-C1 region, but not with Nox-C2 (Fig. 3A). To further map the interaction interface, CRAV was divided into two fragments: the N-terminal 205 amino acids, which overlap with the parental Zfh1 protein, and the C-terminal 69 amino acids, which are unique to CRAV and have undergone rapid evolutionary diversification in *Drosophila* species (46). Co-IP assays demonstrated that only the C-terminal fragment of CRAV interacted with Nox-C1, while the N-terminal fragment showed no binding (Fig. 3B and C).

We next examined whether the CRAV-Nox interaction impacts ROS generation. Overexpression of Nox alone led to increased ROS levels in S2 cells, and this effect was significantly amplified by co-expression of CRAV (Fig. 3D). Importantly, the CRAV C-terminal fragment that interacted with Nox-C1 was sufficient to enhance ROS production, whereas the N-terminal fragment failed to do so (Fig. 3E). These findings indicate that the unique C-terminal domain of CRAV physically associates with the Ca²^+^-binding regulatory domain of Nox to stimulate oxidase activity and synergistically promote ROS generation.

### CRAV-mediated antiviral activity depends on Nox-derived moderate ROS signaling

**Fig 4:**
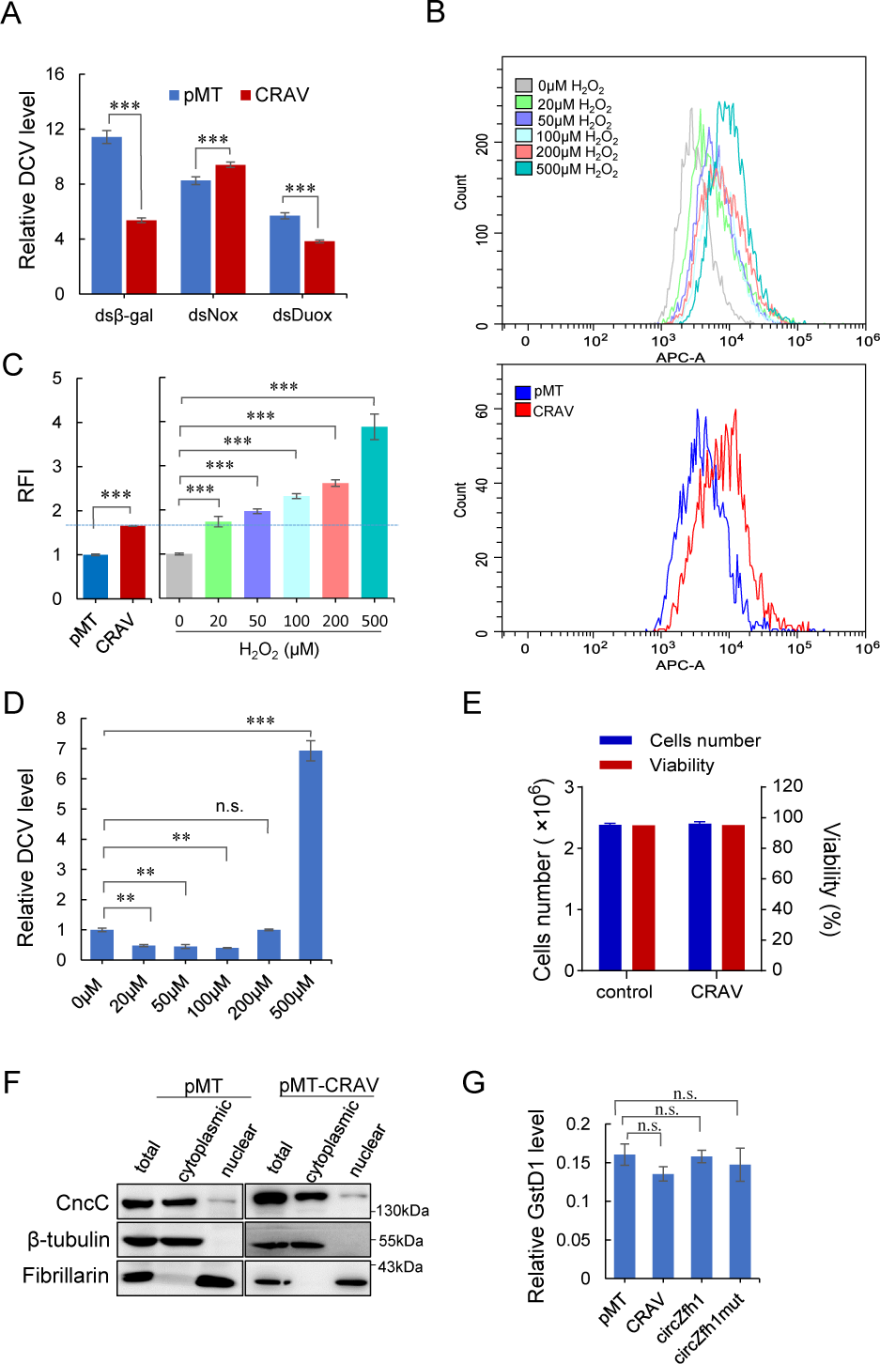
CRAV-mediated antiviral activity depends on Nox-derived moderate ROS signaling. (**A**) S2 cells pretreated with dsRNAs targeting β-gal (control), Nox, or Duox were transfected with pMT or pMT-CRAV and infected with DCV. Viral RNA levels normalized to rp49 were measured by RT-qPCR at 48 h post-infection (hpi). (**B**) Flow cytometry analysis of intracellular ROS levels in S2 cells treated with increasing concentrations of H₂O₂ for 1 h (top) or transfected with pMT or pMT-CRAV (bottom). Overlay histograms show the fluorescence intensity. (**C**) Bar graph quantification of relative fold changes in ROS levels from (**B**). (**D**) S2 cells pretreated with different concentrations of H₂O₂ for 1 h were infected with DCV, and viral RNA levels normalized to *rp49* were analyzed by RT-qPCR at 48 hpi. (**E**) Cell viability assay of S2 cells transfected with pMT or pMT-CRAV for 48 h. Cells were stained with Trypan blue, and live (unstained) and dead (blue) cells were counted microscopically. Viability was calculated as live cells/(live + dead cells). (**F**) Western blot analysis of cytoplasmic and nuclear fractions of S2 cells transfected with pMT or pMT-CRAV, probed with anti-CncC, β-tubulin (cytoplasmic marker), and fibrillarin (nuclear marker). CncC, the *Drosophila* homolog of mammalian Nrf2, translocates to the nucleus when the ARE pathway is activated. (**G**) RT-qPCR analysis of GstD1 expression, a canonical ARE pathway marker, in S2 cells transfected with pMT, pMT-CRAV, pMT-circZfh1, or pMT-circZfh1mut. Data represent mean ± SD from three independent experiments (**A, C–E, G**). Statistical significance was determined by two-way ANOVA (A), one-way ANOVA (**C, D, G**), Student’s *t*-test (**E**): **P < 0.01; ***P < 0.001; n.s., not significant. The representative of triplicate experiments is shown in (**F**).

To investigate whether ROS are required for the antiviral activity of CRAV, we first examined the contribution of NADPH oxidases. Consistent with our earlier findings that CRAV upregulates intracellular ROS via Nox (Fig. 2C and D; Fig. S1F), we compared CRAV-induced resistance to DCV following dsRNA-mediated knockdown of Nox or Duox. In control *β-gal*-dsRNA-treated S2 cells, CRAV expression significantly reduced DCV replication (Fig. 4A). Similarly, CRAV retained antiviral activity in *Duox*-knockdown cells, although DCV levels were already markedly reduced by *Duox* depletion alone. This observation suggests that Duox, which contributes to redox homeostasis in flies, may influence basal viral replication indirectly by shaping the cellular oxidative environment. Importantly, CRAV expression still further suppressed DCV replication in *Duox*-deficient cells, indicating that its antiviral activity is not dependent on Duox (Fig. 4A). By contrast, Nox knockdown yielded a distinct outcome. As with Duox, Nox depletion alone reduced DCV replication, consistent with redox imbalance being unfavorable for viral propagation. However, under these conditions, CRAV expression no longer conferred antiviral protection. Instead, DCV replication was slightly elevated compared with *Nox*-knockdown cells lacking CRAV (Fig. 4A). These findings demonstrate that while both Nox and Duox influence the redox state and thereby modulate baseline susceptibility to viral infection, only Nox is specifically required for the antiviral function of CRAV.

To further define the relationship between ROS levels and DCV replication, we manipulated intracellular redox states by treating S2 cells with hydrogen peroxide (H₂O₂). Increasing concentrations of H₂O₂ induced a dose-dependent increase in cellular ROS (Fig. 4B and C). Strikingly, DCV replication displayed a biphasic response: low concentrations of H₂O₂ suppressed viral replication, whereas high concentrations enhanced it (Fig. 4D). Exogenous expression of CRAV elevated ROS to a level comparable to that induced by low-dose H₂O₂ (Fig. 4B and C), correlating with reduced DCV replication. These data indicate that CRAV promotes an antiviral state by inducing a moderate, Nox-dependent ROS signal, whereas excessive ROS disrupts cellular homeostasis and paradoxically favors viral replication. Thus, CRAV appears to fine-tune redox signaling by engaging Nox to generate moderate ROS that are optimal for antiviral defense.

### CRAV-induced moderate ROS does not elicit cellular oxidative stress

During virus-host interactions, ROS exert biphasic effects, with moderate levels acting as signaling mediators that activate antiviral pathways such as JAK-STAT and NF-κB, while excessive ROS cause oxidative damage, disrupt mitochondrial and endoplasmic reticulum function, and suppress immune signaling, thereby creating conditions that favor viral replication and spread (19, 54–56). Given this dual role, we next asked whether the ROS induced by CRAV activate cellular antioxidant defenses, which are typically engaged under conditions of oxidative stress.

Examination of CRAV-expressing S2 cells revealed that CRAV-induced upregulation of intracellular ROS did not cause cytopathic effects or cell death (Fig. 4E), suggesting that the increase in ROS remained within a physiological range. We therefore tested whether CRAV triggers the antioxidant response element (ARE) pathway, a conserved mechanism regulated by the transcription factor Nrf2. Under oxidative stress, Nrf2 translocates from the cytoplasm to the nucleus, where it binds ARE motifs in the promoters of detoxification and cytoprotective genes, including the glutathione S-transferase family (57). To address this, we performed nuclear-cytoplasmic fractionation of S2 cells transfected with CRAV. Western blot analysis showed that CncC, the *Drosophila* homolog of mammalian Nrf2, remained in the cytoplasm and did not translocate into the nucleus (Fig. 4F). Consistently, the expression of GstD1, a canonical ARE pathway marker, was unaffected by CRAV expression (Fig. 4G). These findings indicate that CRAV-induced ROS do not activate the ARE pathway and are therefore unlikely to represent severe oxidative stress.

### CRAV-induced moderate ROS activates the ASK1-p38 MAPK signaling axis

Our results showed that CRAV expression elevates intracellular ROS to moderate levels without triggering severe oxidative stress or cytotoxicity (Fig. 1D and 4E through G). Such moderate ROS are increasingly recognized as signaling mediators that activate antiviral pathways rather than damaging host cells. In mammals, diverse DNA and RNA viruses activate MAPK cascades, including the p38 pathway, leading to the induction of proinflammatory cytokines (e.g., IL-1β and IL-6) and antiviral effectors (44). This activation can occur through membrane-bound pattern recognition receptors (e.g., TLRs) sensing viral components, through direct interactions of viral proteins with host kinases or via ROS generated by NADPH oxidases (44, 58, 59). Importantly, p38 signaling is conserved in *Drosophila*, where it has been implicated in stress responses and innate antiviral immunity (40, 45, 60).

**Fig 5:**
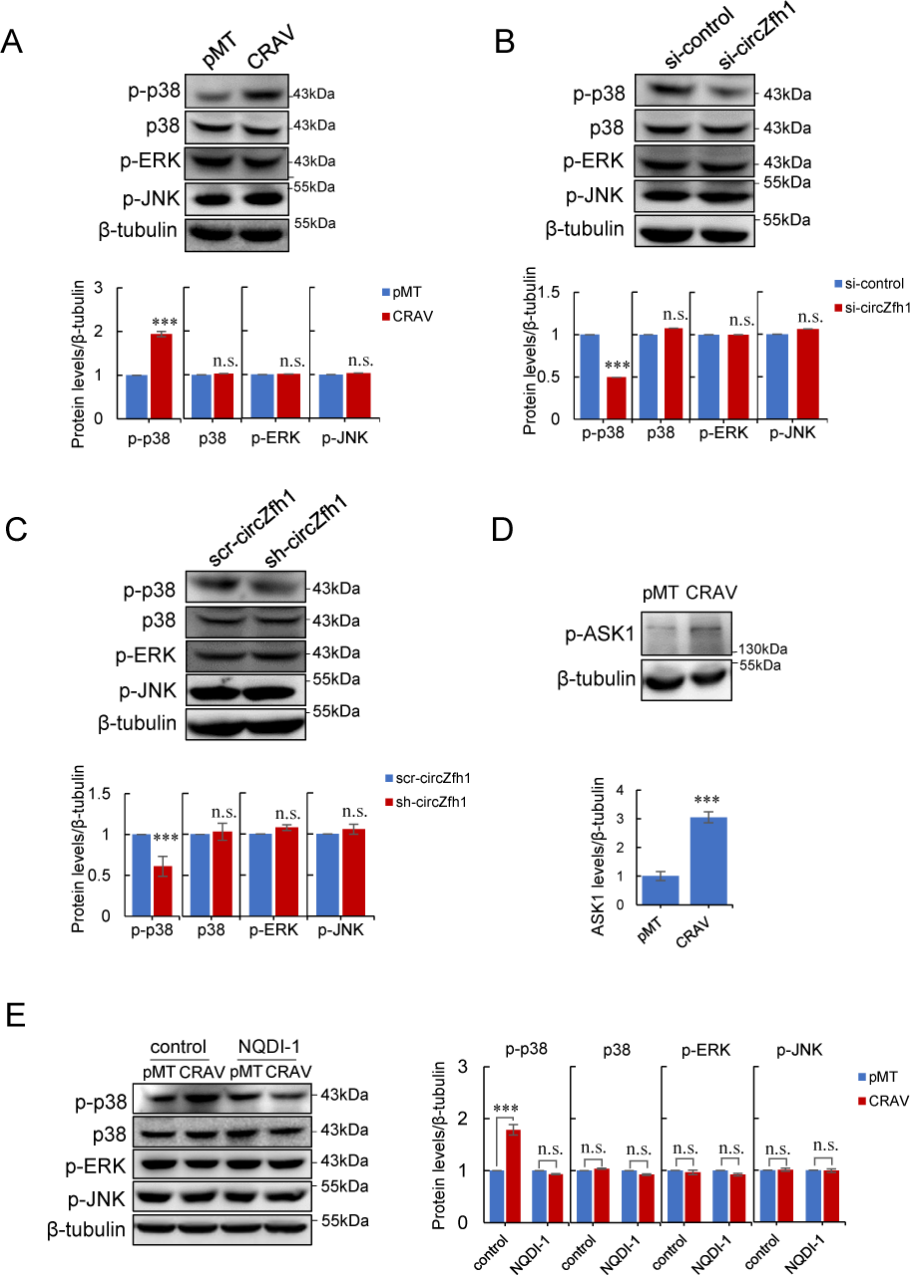
CRAV-induced moderate ROS activates the ASK1–p38 MAPK signaling axis. (**A–C**) Western blot analysis of p-p38, p38, p-ERK, and p-JNK in S2 cells transfected with pMT or pMT-CRAV. (**A**) S2 cells transfected with si-control or si-circZfh1 (**B**) or scr-circZfh1 or sh-circZfh1 (C). The bar graph indicates the relative fold change calculated from the average protein levels, normalized to β-tubulin. (**D**) Western blot analysis of p-ASK1 in S2 cells transfected with pMT or pMT-CRAV. The bar graph indicates the relative fold change calculated from the average protein levels, normalized to β-tubulin. (**E**) Western blot analysis of p-p38, p38, p-ERK, and p-JNK in S2 cells pretreated with DMSO (control) or 10 μM NQDI-1 for 1 h and transfected with pMT or pMT-CRAV. The bar graph indicates the relative fold change calculated from the average protein levels, normalized to β-tubulin. Mean ± SD of three independent experiments is shown (**A–E**); statistical analysis was performed using Student’s *t*-test (**A–D**) and two-way ANOVA (**E**) : *** *P* < 0.001; n.s., not significant. The representatives of triplicate experiments are shown in (**A–E**).

To test whether CRAV-induced ROS activates MAPK signaling in *Drosophila* S2 cells, we examined the phosphorylation status of the three major MAPKs (p38, JNK, and ERK). Western blot analysis revealed that CRAV expression selectively increased phosphorylated p38 (p-p38) levels, while total p38, p-JNK, and p-ERK remained unchanged (Fig. 5A). Moreover, depletion of circZfh1, the precursor of CRAV, reduced p-p38 levels without affecting JNK or ERK phosphorylation (Fig. 5B and C), indicating that CRAV specifically promotes p38 activation. ASK1 (apoptosis signal-regulating kinase 1) is a conserved MAP3K that senses ROS and activates both JNK and p38 cascades (61). We found that CRAV expression increased phosphorylation of ASK1 (Fig. 5D), and importantly, the ASK1 inhibitor NQDI-1 (62) abrogated CRAV-induced p38 phosphorylation without altering JNK or ERK activity (Fig. 5E). Together, these findings demonstrate that CRAV-induced moderate ROS specifically activate the ASK1-p38 MAPK axis, a conserved antiviral pathway, while leaving JNK and ERK pathways unaffected.

### CRAV-induced ROS activate the ASK1-p38-JAK-STAT signaling cascade to confer antiviral protection

In *Drosophila*, activation of the p38 MAPK pathway induces the cytokine upd3, which subsequently stimulates the JAK-STAT pathway via the receptor Domeless, driving the expression of downstream effectors such as TotA and TotM that promote host defense against infection (40, 63, 64). We previously demonstrated that CRAV expression enhances immune responses against DCV infection by upregulating TotA and TotM, indicating that CRAV can activate the JAK-STAT pathway (46). Given our findings that CRAV induces moderate ROS through Nox, we hypothesized that Nox-derived ROS activate the ASK1-p38-JAK-STAT axis to confer antiviral protection.

**Fig 6:**
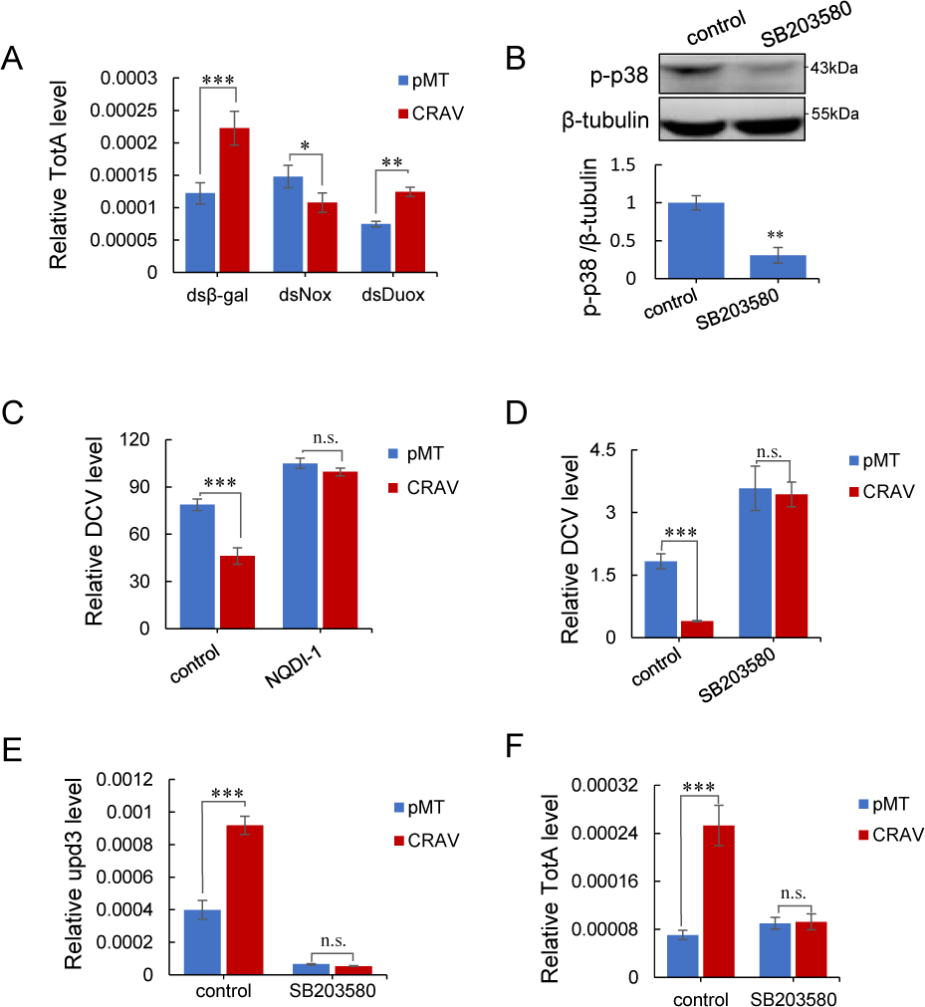
CRAV-induced ROS activate the ASK1-p38-JAK-STAT signaling cascade to confer antiviral protection. (**A**) S2 cells were pretreated with dsRNAs against a control (*β-gal*) or *Nox* and *Duox* and transfected with pMT or pMT-CRAV, followed by RT-qPCR to determine the relative mRNA levels of TotA. (**B**) S2 cells were pretreated with 10 μM SB203580 or DMSO (control) for 1 h, followed by Western blot analysis for p-p38. The bar graph indicates the relative fold change calculated from the average protein levels, normalized to β-tubulin. (**C**) S2 cells pretreated with DMSO (control) or 10 μM NQDI-1 for 1 h and transfected with pMT or pMT-CRAV were infected with DCV. The viral RNA levels at 48 hpi, normalized to rp49, were determined by RT-qPCR. (**D**) S2 cells pretreated with 10 μM SB203580 or DMSO (control) for 1 h and transfected with pMT or pMT-CRAV were infected with DCV. The viral RNA levels at 48 hpi, normalized to rp49, were determined by RT-qPCR. (**E**) S2 cells were pretreated with 10 μM SB203580 or DMSO (control) for 1 h and transfected with pMT or pMT-CRAV, followed by RT-qPCR to determine the relative mRNA levels of upd3 normalized to rp49. (**F**) S2 cells were pretreated with 10 μM SB203580 or DMSO (control) for 1 h and transfected with pMT or pMT-CRAV, followed by RT-qPCR to determine the relative mRNA levels of TotA normalized to rp49. The mean ± SD of three independent experiments is shown (**A–F**); statistical analysis was performed using two-way ANOVA (**A, C–F**) and Student’s *t*-test (**B**): * *P* < 0.05; ** *P* < 0.01; *** *P* < 0.001; n.s., not significant. The representative of triplicate experiments is shown in (**B**).

To test this hypothesis, we first examined whether CRAV-induced JAK-STAT activation depends on NADPH oxidase-derived ROS. Indeed, knockdown of Nox, but not Duox, suppressed CRAV-mediated induction of TotA (Fig. 6A), consistent with our earlier findings that CRAV requires Nox-derived ROS to confer antiviral protection. We next investigated whether the ASK1-p38 module functions downstream of CRAV. Pharmacological inhibition of p38 using SB203580 (which specifically blocks p38 without affecting JNK or ERK in *Drosophila* [65]), markedly reduced p38 phosphorylation in S2 cells (Fig. 6B). Importantly, CRAV expression no longer conferred resistance to DCV infection under either NQDI-1 or SB203580 treatment (Fig. 6C and D), indicating that ASK1-p38 signaling is indispensable for CRAV-mediated antiviral protection.

Finally, we directly tested whether CRAV-induced activation of the JAK-STAT pathway depends on p38 activity. Inhibition of p38 by SB203580 blocked CRAV-driven upregulation of both upd3 and TotA (Fig. 6E and F). These findings demonstrate that p38 signaling is required for JAK-STAT pathway activation downstream of CRAV. Together, these results establish that CRAV engages Nox-derived ROS to activate the ASK1-p38 MAPK cascade, which in turn induces JAK-STAT signaling, thereby restricting DCV replication.

## DISCUSSION

In this study, we demonstrate that the circular RNA-encoded peptide CRAV confers antiviral immunity by binding the NADPH oxidase Nox to induce a moderate level of ROS. These ROS act as signaling molecules to specifically activate the ASK1-p38 MAPK axis, which in turn triggers the JAK-STAT pathway and antiviral gene expression, revealing a crucial role for finely tuned ROS signaling in host defense.

Viral pathogens frequently disrupt cellular redox balance either by elevating ROS as a consequence of replication or by directly manipulating host oxidase systems (21–23, 58, 59). The consequences are highly context-dependent: moderate, localized ROS enhance antiviral responses, whereas uncontrolled oxidative stress undermines host defenses and promotes persistence (19, 21–28). In this study, our gain- and loss-of-function analyses show that CRAV is both necessary and sufficient to elevate ROS levels and that this increase arises specifically from Nox-dependent NADPH oxidase activity, rather than from mitochondrial dysfunction. Notably, CRAV-induced ROS do not trigger Nrf2/ARE activation, cytopathic effects, or oxidative stress markers, indicating that CRAV generates a controlled, signaling-competent redox output (Fig. 4E through G). This is consistent with extensive evidence showing that ROS function as versatile signaling intermediates in both insects and mammals (19, 20). Among downstream effectors, MAPK cascades act as conserved redox-sensitive modules, with p38 serving as a central hub that links oxidative signals to cytokine production (40, 43–45). CRAV fits into this framework by directly interacting with the Ca²^+^-binding regulatory domain of Nox, enhancing ROS production in a controlled manner (Fig. 4A and D). These ROS activate ASK1, a redox-sensitive MAP3K, which in turn selectively phosphorylates p38 without engaging JNK or ERK (Fig. 5E). Although *Drosophila* encode three p38 isoforms (p38a, p38b, and p38c), distinguishing which isoform acts downstream of CRAV will require future investigation. This controlled modulation of ROS highlights a novel layer of antiviral regulation, where host-encoded proteins exploit redox biology to engage immune pathways.

Our findings further reveal that CRAV activates an ROS-ASK1-p38-Upd3-JAK-STAT cascade that provides antiviral protection against DCV infection in *Drosophila*. In flies, JAK-STAT is an evolutionarily conserved pathway with developmental and immune functions. Activation begins when the cytokine Unpaired (Upd) binds the receptor Domeless, leading to STAT92E phosphorylation and induction of effector genes such as TotA, TotM, and socs36E (40, 63, 64). Although RNAi is the dominant antiviral mechanism, JAK–STAT provides a critical backup when RNAi is suppressed by VSRs (46). CRAV mechanistically links ROS to JAK-STAT activation: by amplifying Nox-derived ROS, CRAV triggers ASK1-p38 signaling, which drives upd3 expression and cytokine release. Inhibition of ASK1 or p38 abolishes CRAV-induced Upd3 secretion and JAK–STAT target gene activation, eliminating its antiviral effect (Fig. 6B through F). Likewise, Nox depletion, but not Duox, suppresses CRAV-driven TotA induction, confirming that Nox-derived ROS function upstream of this cascade (Fig. 6A).

This ROS-ASK1-p38-Upd3-JAK-STAT pathway is conceptually significant for several reasons. First, it shows that circRNA-derived proteins can reprogram host immunity by engaging conserved stress pathways. Second, it demonstrates that controlled ROS generation is sufficient to activate cytokine-mediated antiviral signaling. Third, it explains why CRAV’s activity is restricted to viruses with intact VSRs (46): by engaging an ROS-MAPK-JAK-STAT axis, CRAV ensures defense specifically when RNAi is neutralized. More broadly, this cascade highlights the modularity of innate immunity. While RNAi provides sequence-specific clearance, the ROS-ASK1-p38-Upd3-JAK-STAT axis acts as a redundant defense, ensuring survival when RNAi is disabled. Comparable redundancy exists in mammals, where interferons, NF-κB, and inflammasomes compensate for one another (66, 67). From an evolutionary perspective, the unique C-terminal extension of CRAV, generated by a frameshift during circRNA translation (46), may represent an adaptive innovation that enables interaction with Nox and modulation of ROS. Its rapid diversification across *Drosophila* species suggests ongoing host-virus coevolution, where novel protein domains in circRNA-derived proteins provide selective advantages against viral challenges.

## MATERIALS AND METHODS

### Plasmid construction

The expression constructs illustrated in Fig. 1C were generated in the pMT/V5-His A vector (Invitrogen, USA, Cat. No. V412020). DNA fragments were amplified from S2 cell genomic DNA and cloned into pMT/V5-His A using the One Step Cloning Kit (Vazyme, Nanjing, China, Cat. No. C115). The plasmid pMT-CRAV was constructed by inserting the linearized full-length ORF of circZfh1. The plasmid pMT-circZfh1 was generated following a previously described protocol (68). To abolish protein translation, the start codon (ATG) at exon 3 of pMT-circZfh1 was mutated to TTG, yielding pMT-circZfh1mut. All constructs were verified by Sanger sequencing. Primers are listed in Table S1.

### Cell culture and virus infection

*Drosophila* S2 cells were maintained in Schneider’s insect medium (Sigma-Aldrich, USA, Cat. No. S9895) with 10% fetal bovine serum (FBS) (Gibco, USA, Cat. No. 10099141), 5 mM NaHCO_3_ (Sigma-Aldrich, USA, Cat. No. S3817), 5 mM CaCl_2_ (Sigma-Aldrich, USA, Cat. No. C7902), and 100 U/mL penicillin and 100 μg/mL streptomycin (HyClone, USA, Cat. No. SV30010) at 25°C.

For virus infection, S2 cells were seeded into 24-well plates and allowed to attach for 3 h at 25°C before infection with DCV at a multiplicity of infection (MOI) of 1. For transfection assays, cells were first transfected with the indicated plasmids and incubated for 24 h, followed by DCV infection (MOI = 1). Viral replication or gene expression levels were determined at the indicated time points by RT-qPCR using the primers listed in Table S1.

### RNA extraction and RT-qPCR

Total RNA was extracted from S2 cells, whole flies, or dissected tissues using TRIzol according to the manufacturer’s instructions. Reverse transcription was performed using the RevertAid First-Strand cDNA Synthesis Kit (Thermo Fisher Scientific, USA, Cat. No. K1622). Real-time PCR was conducted on a LightCycler 96 System (Roche, Switzerland) with ChamQ Universal SYBR qPCR Master Mix (Vazyme, Nanjing, China, Cat. No. Q311) following the manufacturer’s instructions. Primers for qRT-PCR are listed in Table S1. All genes were assayed in triplicate, and relative expression to rp49 was calculated with the 2-ΔΔCt method (69).

### Plasmid DNA transfection

For the transfection experiment, S2 cells were plated in 12-well plates and grown for several hours to reach 80% confluence. Then, 1 μg of the plasmid was transfected into the cells using Lipofectamine 6000 Transfection Reagent (Beyotime, Shanghai, China, Cat. No. C0526) according to the manufacturer’s protocol. The cells were stimulated with 25 μM CuSO_4_ at 24 h post-transfection to induce protein expression driven by the pMT promoter. Transfected cells were harvested and directly processed to extract total RNA or protein at 2 days post-transfection.

### RNA sequencing data analysis

First, ribosomal RNAs and other contaminants were removed from raw sequencing reads. The remaining clean reads were mapped to the *Drosophila* genome (dm6 assembly) using STAR (70), allowing no more than 4 mismatches per read pair and no more than 20 multiple mapping loci. Gene expression levels in the form of fragments per kilobase of exon model per million mapped fragments (FPKM) were calculated by Cuffdiff2 (71) based on gene annotation provided by Flybase (version r6.04). Differential gene expression analysis was performed by Cuffdiff2 (71), and genes with fold change > 2 (*P* value< 0.05) and > 1 FPKM value in at least one sample were regarded as DEGs. To gain insight into the possible functions of the selected DEGs, we conducted GO analysis using the clusterProfiler package (version 4.2.2) in R (version 4.1.0) (72). GO analysis involves annotations in three distinct categories: biological process, cellular component, and molecular function. A *P* value < 0.05 was considered to indicate statistical significance. Bubble plots were generated using the R package ggplot2 (version 3.3.5).

### Gene knockdown by RNAi

For dsRNA-based RNAi, the protocol followed the guidelines provided at https://fgr.hms.harvard.edu. DNA templates for dsRNA synthesis were obtained by PCR using gene-specific primers that contained the T7 polymerase recognition sequence at their 5′ end. The primer sequences can be found in Table S1. The dsRNAs targeting each gene were synthesized using a T7 transcription kit (Toyobo, Japan, Cat. No. TSK-101) and visualized by agarose gel electrophoresis. Approximately 1.5 × 10^6^ S2 cells were bathed in 400 μL of serum-free medium containing 7 μg of dsRNA per well of a 12-well plate for 30 min at 25°C. The cells were then supplemented with 400 μL of complete medium containing 20% FBS and incubated at 25°C. After 2 days, the cells were harvested to assess the knockdown efficiency or used for virus infection.

For siRNA-based RNAi, S2 cells were plated in 12-well plates and allowed to reach 80% confluence. Then, 8 pmol of siRNA was transfected into the cells using Lipofectamine 6000 Transfection Reagent following the manufacturer’s protocol. After culturing at 25°C for 2 days, the cells were harvested to assess the knockdown efficiency or used for virus infection. The sequences of siRNAs targeting circRNAs can be found in Table S1.

### RNA-seq library construction and sequencing

For the transcriptomic analysis shown in Fig. 1A and B, total RNAs were isolated from S2 cells transfected with pMT-CRAV or pMT (pMT/V5-His A) plasmid for 48 h using TRIzol. The transcriptome libraries were constructed and sequenced using BGISEQ-500 at Genewiz (Suzhou, China).

### Western blot analysis

S2 cells from a single well of a 12-well plate were harvested and washed twice with ice-cold PBS buffer. Cell lysis was performed by adding 80 μL of cold lysis buffer (20 mM Tris-HCl pH 7.0, 50 mM NaCl, 0.5 mM EDTA, 0.5% NP40, 0.5% sodium deoxycholate, and 1 mM PMSF) and keeping the samples on ice for 60 min. The cell extracts were then centrifuged at 12,000 rpm for 15 min at 4°C, and the supernatants were collected as total protein extracts. The protein extracts were separated by SDS-PAGE and transferred to a PVDF membrane (Millipore, USA, Cat. No. IPVH00010). The membrane was blocked and then incubated with the designated primary antibodies overnight at 4°C. After washing, the membranes were incubated with HRP-labeled goat anti-mouse/rabbit IgG secondary antibody (Beyotime, Shanghai, China, Cat. No. A0216/Cat. No. A0208) for 1 h. Immunoreactivity was detected using ECL substrates (Beyotime, Shanghai, China, Cat. No. P0018) and analyzed with LAS4000 (GE Healthcare, USA). Quantification of the Western blot bands was performed using ImageJ software (http://rsb.info.nih.gov/ij/index.html).

In this study, the following commercial antibodies were used: anti-β-tubulin (1:5,000, CWbio, Jiangsu, China, Cat. No. CW0098, RRID: AB_2814800), anti-V5 (1:10,000, Invitrogen, USA, Cat. No. R960-25, RRID: AB_2556564), anti-Flag (1:12,000, Sigma-Aldrich, USA, Cat. No. F3165, RRID: AB_259529), anti-fibrillarin (1:1,000, Abcam, USA, Cat. No. ab5821, RRID: AB_2105785), anti-p-p38 (1:500, ZENBIO, Chengdu, China, Cat. No. 310091, RRID: AB_2938885), anti-p38 (1:500, ZENBIO, Chengdu, China, Cat. No. R25239, RRID: AB_2938895), anti-p-ERK (1:500, ZENBIO, Chengdu, China, Cat. No. 310065, RRID: AB_2938897), anti-p-JNK (1:500, ZENBIO, Chengdu, China, Cat. No. 340810, RRID: AB_2938898), and anti-p-ASK1 (1:500, ZENBIO, Chengdu, China, Cat. No. 320030, RRID: AB_2938899).

### Subcellular fractionation

The subcellular fractionation protocol was followed as previously described (73). Briefly, 1 × 10^8^ S2 cells were lysed with hypotonic buffer (30 mM HEPES pH 7.4, 2 mM MgOAc, 5 mM DTT, 0.2% NP-40, and 1 mM PMSF) and homogenized using a 30G needle. The lysate was then centrifuged at 500 × *g* for 20 min, and the resulting supernatant was collected as the cytoplasmic fraction. The pellet was washed four times with hypotonic buffer and then lysed in nuclear lysis buffer (20 mM Tris-HCl pH 7.0, 50 mM NaCl, 0.5 mM EDTA, 0.5% NP40, 0.5% sodium deoxycholate, and 1 mM PMSF), followed by centrifugation at 12,000 rpm for 15 min to obtain the supernatant as the nuclear fraction. Equal amounts of protein from each lysate were subjected to Western blot analysis. All fractionation steps were performed at 4°C or on ice.

### Immunoprecipitation

S2 cells were transfected with the relevant plasmids and cultured for 48 h. Transfected cells were collected and washed with PBS. The cells were then lysed in cell lysis buffer (50 mM Tris HCl pH 7.4, 150 mM NaCl, 1 mM EDTA, 1% Triton X-100, and 1 mM PMSF) on ice for 1 h. After centrifugation at 12,000 rpm for 15 min at 4°C, the lysates were incubated overnight at 4°C with either Anti-Flag M2 Affinity Gel (Sigma-Aldrich, USA, Cat. No. A2220) or Protein A/G Agarose beads (Santa Cruz, USA, Cat. No. sc-2003) conjugated with anti-V5 antibody (Invitrogen, USA, Cat. No. R960-25, RRID: AB_2556564). The immunoprecipitates were then washed five times with cell lysis buffer and subjected to Western blot analysis.

### Intracellular ROS measurement

Intracellular ROS were measured using CellROX Oxidative Stress Reagent (Invitrogen, USA, Cat. No. C10422) by flow cytometry following the manufacturer’s instructions. Briefly, 1 × 10^6^ cells were treated with 5 μM CellROX Reagent at 37°C for 30 min. The labeled cells were then washed with PBS and evaluated immediately by flow cytometry (Beckman Coulter CytoFLEX, USA). All experimental steps were performed in the dark to minimize potential light-induced artifacts.

### Measurement of mitochondrial membrane potential

A total of 5 × 10^5^ cells were stained with JC-1 solution (Beyotime, Shanghai, China, Cat. No. C2006) for 20 min at 37°C, washed twice with PBS, and analyzed using a fluorescence spectrophotometer (HITACHI F-4600, Japan).

### Statistical analysis

Each experiment was repeated independently at least three times with similar results. The Western blotting images are representative of three independent experiments. Unless stated otherwise, the results of quantitative experiments are reported as mean ± SD of three independent experiments. Error bars represent SD. Statistical analysis was performed with Prism Version 10.3 (GraphPad). For comparisons involving two independent variables, two-way ANOVA followed by Sidak’s multiple comparisons test was applied. For comparisons of multiple groups against a single control group, one-way ANOVA was performed, followed by Dunnett’s multiple comparisons test. Two-tailed unpaired (Student’s) *t*-test was performed if only two conditions were compared. Statistical significance was defined as follows: n.s., not significant; * *P* < 0.05; ** *P* < 0.01; and *** *P* < 0.001.

## Data Availability

The data sets for pMT/pMT-CRAV-expressing and DCV-infected S2 cells are available in the NCBI SRA database (Sequence Read Archive: https://www.ncbi.nlm.nih.gov/sra), with the accession numbers PRJNA960708 and PRJNA960676, respectively.

## SUPPLEMENTAL MATERIAL

Supplemental material jvi.01708-25-s0001.pdf Figure S1 and Table S1.

ASM does not own the copyrights to Supplemental Material that may be linked to, or accessed through, an article. The authors have granted ASM a non-exclusive, world-wide license to publish the Supplemental Material files. Please contact the corresponding author directly for reuse.
