# Supplementary material for: CircRNA-encoded protein fine-tunes ROS homeostasis and engages conserved JAK-STAT antiviral defenses in Drosophila
Source: J Virol. 2025 Dec 22;100(1):e01708-25. doi: 10.1128/jvi.01708-25 (PMC12817946; doi:10.1128/jvi.01708-25)
Supplement: Supplemental material — Figure S1 and Table S1. [file jvi.01708-25-s0001.pdf]

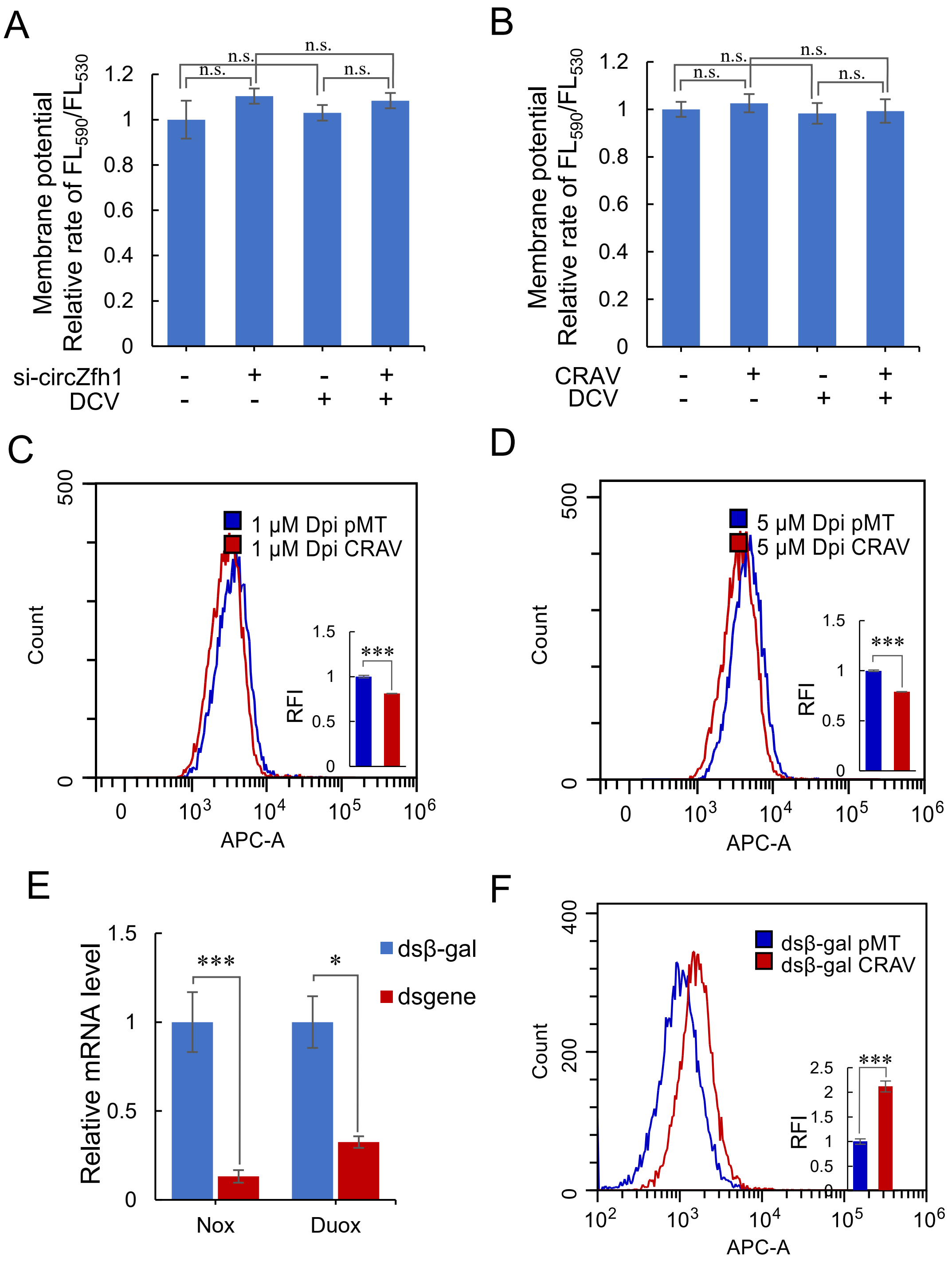

Fig. S1. CRAV-induced ROS production is mediated by Nox.

(**A**) Mitochondrial membrane potential was measured in S2 cells with or without DCV infection and transfected with si-control or si-circZfh1. (**B**) Mitochondrial membrane potential was measured in S2 cells with or without DCV infection and transfected with pMT or pMT-CRAV. (**C-D**) Detection of cellular ROS levels by flow cytometry in S2 cells pretreated with 1 μM Dpi (**C**) or 5 μM Dpi (**D**) for 1 h and transfected with pMT or pMT-CRAV. (**E**) S2 cells were pretreated with dsRNAs against a control (β-gal) or Nox, Duox, followed by RT-qPCR analysis of mRNA levels of indicated genes relative to the control. (**F**) Detection of cellular ROS levels by flow cytometry in S2 cells pretreated with dsRNAs against β-gal and transfected with pMT or pMT-CRAV. The peak plot indicates the fluorescence intensity of the indicated cells, and the bar graph indicates the relative fold change calculated from the average fluorescence intensity values(**C**,**D**,**F**). Mean ± SD of three independent experiments is shown (A-F); statistical analysis was performed using two-way ANOVA (A-B), Student's t-test (C-F): * $p < 0.05$; *** $p < 0.001$; n.s., not significant.

Supplementary Table 1: Primers and sequences

Sequences of primers used in plasmid construction

primer name	sequence(5'-3')
pMT-circZfh1-Fw	AGGGGGGATCTAGATCGGGGTACCACAGCCCTCAGATACAGATACT
pMT-circZfh1-Rv	CGGCCGCCACTGTGCTGGATATCTTCGGTCCGGTGAACGCCTACG
pMT-CRAV-Fw	TCTAGATCGGGGTACCATGAGCGGCAGCAGCAGGCGGT
pMT-CRAV-Rv	GATATCTGCAGAATTCTCAGCTTGTCGTGGGGATGCTC
pMT-circZfh1mut-Fw	ACTTGAGCGGCAGCAGCAGGCGGT
pMT-circZfh1mut-Rv	TGCTGCCGCTCAAGTTGCTCTGCT
pMT-Nox-C1-V5-Fw	GGGGATCTAGATCGGGGTACCATGAACGCGGACCAGGAGTCGAACA
pMT-Nox-C1-V5-Rv	TGCTGGATATCTGCAGAATTCCACCTGGTTGTTCTTCATGTAG
pMT-Nox-C2-V5-Fw	GGGGATCTAGATCGGGGTACCATGAGGGGAGAGCACGGCAAGACGTACA
pMT-Nox-C2-V5-Rv	TGCTGGATATCTGCAGAATTCGAAGCACTCCTTACGAAAGGCAAAT
pMT-205aa-Fw	GGGGGGATCTAGATCGGGGTACCATGAGCGGCAGCAGCAGGCGGT
pMT-205aa-Rv	TGTGCTGGATATCTGCAGAATTCCTGGGAGACATTGCTGACCGC
pMT-69aa-Fw	GGGGATCTAGATCGGGGTACCATGTTCCCTCGCTGGCCTCCACTTT
pMT-69aa-Rv	TGCTGGATATCTGCAGAATTCGCTTGTCGTGGGGATGCTCGCT

Sequences of primers used in circZfh1 knockdown

circZfh1 siRNA Fw	CAAUGUCUCCCAGUUCCCUTT
circZfh1 siRNA Rv	AGGGAACUGGGAGACAUUGTT
circZfh1 si-control Fw	ACGCUUCUACCGUACUCCUTT
circZfh1 si-control Rv	AGGAGUACGGUAGAAGCGUTT
circZfh1-shRNA-F	cagtCAATGTCTCCCAGTTCCCTCGtagttatattcaagcataCGAGGGAACTGGGAGACATTGgcg
circZfh1-shRNA-R	aattcgcCAATGTCTCCCAGTTCCCTCGtatgcttgaatataactaCGAGGGAACTGGGAGACATTGactGGTAC
circZfh1- scramble-F	cagtCGACGCTTCTACCGTACTCCTtagttatattcaagcataAGGAGTACGGTAGAAGCGTCGgcg
circZfh1- scramble-R	aattcgcCGACGCTTCTACCGTACTCCTtatgcttgaatataactaAGGAGTACGGTAGAAGCGTCGactGGTAC

Sequences of primers used in RNAi knockdown

primer name	sequence(5'-3')
dsNox-Fw	TAATACGACTCACTATAGGGATACCGCCTGATTGAGTTGG
dsNox-Rv	TAATACGACTCACTATAGGGTAACATCGTGCAGCTCCTTG
dsDuox-Fw	TAATACGACTCACTATAGGGGGATTGGAGCGATGAGGATA
dsDuox-Rv	TAATACGACTCACTATAGGGCCGCACATCTGAACAGAGAA

Sequences of primers used in qPCR

primer name	sequence(5'-3')
rp49-qFw	AGCATACAGGCCCAAGATCG
rp49-qRv	TGTTGTCGATACCCTTGGGC
DCV-qFw	TTTAGCAGATGATGCGGCA
DCV-qRv	GGAGACCAATTTGAGTCGATC
upd3-qFw	AGCCGGAGCGGTAACAAAA
upd3-qFv	CGAGTAAGATCAGTGACCAGTTC
TotA-qFw	CCCAGTTTGACCCCTGAG
TotA-qRv	GCCCTTCACACCTGGAGA
Nox-qFw	CCAATCGCTTGTACCGCTAC
Nox-qRv	CGTTGCTGATCTTTCTCCGG
Duox-qFw	GGACCGATGGAATTCACACG
Duox-qRv	GACTCCCTCGCGGTATTGTA
GstD1-qFw	CAATCCCCAGCACACCATTC
GstD1-qRv	TTGGGGCACTTAGGGTACAG
